# Prediction of biological age and all-cause mortality by 12-lead electrocardiogram in patients without structural heart disease

**DOI:** 10.1186/s12877-021-02391-8

**Published:** 2021-08-11

**Authors:** Naomi Hirota, Shinya Suzuki, Takuto Arita, Naoharu Yagi, Takayuki Otsuka, Takeshi Yamashita

**Affiliations:** Department of Cardiovascular Medicine, The Cardiovascular Institute, 3-2-19 Nishiazabu, Minato-Ku, Tokyo, 106-0031 Japan

**Keywords:** Electrocardiogram, Biological age, All-cause death, Cardiovascular death

## Abstract

**Background:**

There is a well-established relationship between 12-lead electrocardiogram (ECG) and age and mortality. Furthermore, there is increasing evidence that ECG can be used to predict biological age. However, the utility of biological age from ECG for predicting mortality remains unclear.

**Methods:**

This was a single-center cohort study from a cardiology specialized hospital. A total of 19,170 patients registered in this study from February 2010 to March 2018. ECG was analyzed in a final 12,837 patients after excluding those with structural heart disease or with pacing beats, atrial or ventricular tachyarrhythmia, or an indeterminate axis (R axis > 180°) on index ECG. The models for biological age were developed by principal component analysis (BA) and the Klemera and Doubal’s method (not adjusted for age [BA_E_] and adjusted for age [BA_EC_]) using 438 ECG parameters. The predictive capability for all-cause death and cardiovascular death by chronological age (CA) and biological age using the three algorithms were evaluated by receiver operating characteristic analysis.

**Results:**

During the mean follow-up period of 320.4 days, there were 55 all-cause deaths and 23 cardiovascular deaths. The predictive capabilities for all-cause death by BA, BA_E_, and BA_EC_ using area under the curves were 0.731, 0.657, and 0.685, respectively, which were comparable to 0.725 for CA (*p* = 0.760, 0.141, and 0.308, respectively). The predictive capabilities for cardiovascular death by BA, BA_E_, and BA_EC_ were 0.682, 0.685, and 0.692, respectively, which were also comparable to 0.674 for CA (*p* = 0.775, 0.839, and 0.706, respectively). In patients aged 60–74 years old, the area under the curves for all-cause death by BA, BA_E_, and BA_EC_ were 0.619, 0.702, and 0.697, respectively, which tended to be or were significantly higher than 0.482 for CA (*p* = 0.064, 0.006, and 0.005, respectively).

**Conclusion:**

Biological age by 12-lead ECG showed a similar predictive capability for mortality compared to CA among total patients, but partially showed a significant increase in predictive capability among patients aged 60–74 years old.

**Supplementary Information:**

The online version contains supplementary material available at 10.1186/s12877-021-02391-8.

## Background

Age is a strong risk factor for mortality. However, chronological age (CA) itself may not be a reliable indicator of functional deterioration because aging can be heterogeneous, with a balance between exposure to damaging properties and resiliency [1, 2]. The concept of biological age was developed to represent the actual status of individual aging. Biological age is estimated as a single variable using complex equations based on multiple biomarkers, which include physical, physiological, or biochemical indicators of individual health status [2–4]. However, a simple, non-invasive, and cost-effective method for estimating biological age is required for its practical use.

Electrocardiography (ECG) is widely used to detect or evaluate the risk of cardiac diseases. ECG parameters can be affected by age, gender, and individual physical conditions [5, 6], especially those involving the circulatory and respiratory systems. The potential mechanisms underlying these effects include changing topography of the heart in relation to the thorax and diaphragm, modification of the various components of the volume conductor (skin, subcutaneous fat, and lung parenchyma), or alterations in cardiac configuration and intracardiac conduction [5, 7].

Aging is also a key factor underlying these electrophysiological and electroanatomical changes [5, 6, 8]. Several studies have utilized ECG to predict biological age or ‘heart age’ [9–11]. A discrepancy between biological age estimated by ECG and the actual CA was also reported [9], which may relate to differences in the physical conditions of the individuals and the presence of cardiovascular diseases (CVDs). Importantly, this concept may be utilized to provide a simple method for screening patients’ health status. Nevertheless, for actual clinical use it would be important to examine the effects of ethnicity on performance of the biological age prediction models [11]. Furthermore, the majority of reported models only examine several representative ECG parameters and with a linear regression model [10, 12], with only one report utilizing artificial intelligence modeling [9].

.In the present study, we developed a prediction model for biological age that incorporated hundreds of automatically-measured ECG parameters assessed using the principal component analysis (PCA) algorithm [13] and the Klemera and Doubal’s method (KDM) [14] from a single-center cohort in a Japanese cardiovascular hospital. The aim of this study was to evaluate the hypothesis that the predictive capability of biological age for mortality was higher than that of CA.

## Methods

### Study population

The Shinken Database [15] includes all new patients visiting the Cardiovascular Institute, a cardiology specialized hospital in an urban area of Tokyo, Japan. This single hospital-based database was established in June 2004 to investigate the prevalence and prognosis of various types of CVDs. To investigate the new appearance of CVDs, patients who visited our hospital but who were not diagnosed with CVDs at baseline were also included in the cohort. Patients have been continually registered into the database annually and the registration is ongoing (32,570 patients have been registered up to March 2018). Foreign travelers and patients with active cancer were excluded because of the difficulty in evaluating long-term follow-up. The patients seen included both local residents and patients referred from other clinics for treatment of CVDs. The attending physicians were all cardiologists or cardiothoracic surgeons.

We used computerized ECG records, which have been available in our database since February 2010. From a total of 32,570 patients in the Shinken Database, we extracted 19,170 patients registered between February 2010 and March 2018. After excluding patients with structural heart diseases (*n* = 4915), patients aged < 20 years old or > 90 years old (*n* = 168), and patients with an index ECG showing an indeterminate axis (R axis > 180°; *n* = 76), pacing beats (*n* = 102), or atrial or ventricular tachyarrhythmia (*n* = 1763), a total of 12,837 patients were included in the present study.

### Data collection at initial visit

After ECG and chest X-ray were performed, cardiovascular status was evaluated using data from an echocardiogram, exercise test, 24-h Holter recording, and blood laboratory tests at the discretion of the attending physician. In addition to gender, age, height, and weight, we collected data on CVDs, including heart failure (New York Heart Association class ≥2), valvular heart disease (moderate or severe stenosis or regurgitation on echocardiogram), coronary artery disease (diagnosed on angiogram or scintigram), hypertrophic and dilated cardiomyopathy (diagnosed on echocardiography or magnetic resonance imaging), congenital heart disease (diagnosed on echocardiography), and history of disabling cerebral infarction or transient ischemic attack (diagnosed on computed tomography or magnetic resonance imaging). Cardiovascular risk factors were defined as hypertension, use of antihypertensive agents, systolic blood pressure ≥ 140 mmHg, or diastolic blood pressure ≥ 90 mmHg; diabetes mellitus, use of oral hypoglycemic agents or insulin, or glycosylated hemoglobin ≥6.5%; dyslipidemia, use of statin or drugs for lowering triglyceride, low-density lipoprotein ≥140 mg/dL, high-density lipoprotein < 40 mg/dL, or triglyceride ≥150 mg/dL; and chronic kidney disease or estimated glomerular filtration rate (eGFR) < 60 mL/min/1.73 m^2^. The eGFR was calculated using the Japanese coefficient for the modified isotope dilution mass spectrometry-traceable 4-variable Modification of Diet in Renal Disease study equation (eGFR = 194 × SCr^− 1.004^ × Age^− 0.287^ × 0.739 [if female]). Body mass index was calculated as weight in kilograms divided by height in meters squared.

### Patient follow-up

The health status and the incidence of cardiovascular events and mortality were maintained in the database via linking to hospital medical records and by prognosis study documents sent yearly to patients who stopped hospital visits or who were referred to other hospitals. In the present study, we included follow-up data until March 2019 and excluded follow-up data of > 3 years after the initial visit to avoid an imbalance of the follow-up period due to the different registration years (between 2010 and 2018).

### Parameters obtained from ECG

The 12-lead ECG was recorded for 10 s in the supine position using an ECG machine (GE CardioSoft v6.71 and MAC 5500 HD; GE Healthcare, Chicago, IL, USA) at a sampling rate of 500 Hz. Data were stored using the MUSE data management system. Automatic analysis of 639 parameters from the computerized raw ECG data was performed by the GE system. Of these parameters, 201 (nine not lead-specific and 192 [16 × 12 leads] lead-specific) were temporally stored datasets that included the relative coordinate points (i.e., the start point of the P-wave) and calculated values similar to the original parameters (i.e., of the corrected QT [QTc] parameters, the QTc calculation [QTc Bazett] was used while the QTc Framingham and QTc Fridercia were excluded). The remaining 438 parameters (six not lead-specific and 432 [36 × 12 leads] lead-specific) were used in the final analysis (Table 1).

**Table 1:** List of ECG parameters used in this study

Parameters available in MUSE database system: 639 parameters	
Parameters used for analysis: 438 parameters	
(1) Not lead-specific parameters: 6 parameters P-R Interval, P axis, QRS Duration, QTc Calculation (QTc Bazett), R axis, T axis (2) Lead-specific parameters: 432 [36 × 12 leads] parameters ST at J Point, P Area, P′ Area, P Area (Full), P Peak Time, P′ Peak Time, P Peak Amplitude, P′ Peak Amplitude, P Duration, P′ Duration, QRS Area, Q Area, Q Peak Amplitude, Q Duration, R Area, R’ Area, R Peak Time, R Duration, R’ Duration, S Area, S′ Area, S Peak Time, S Duration, S′ Duration, T Area, T’ Area, T Area (Full), T Peak Time, T Peak Amplitude, T’ Peak Amplitude, T Duration, T’ Duration, Minimum ST level, Max R Amplitude, Maximum ST level, Max S Amplitude	
Parameters excluded: 201 parameters	
(1) Not lead-specific parameters: 9 parameters P Onset, P Offset, QRS Count, QTc Framingham, QTc Fridercia, Q-T Interval, Q Onset, Q Offset, T Offset (2) Lead-specific parameters: 192 [16 × 12 leads] parameters P Onset Amplitude, QRS Balance, QRS Deflection, QRS Intrinsicoid, Q Peak Time, R’ Peak Time, R Peak Amplitude, R’ Peak Amplitude, S′ Peak Time, S Peak Amplitude, S′ Peak Amplitude, T’ Peak Time, T End, ST at End ST, ST at Mid ST, Special T	

P′, R’, S′, and T’ indicated the second components of P, R, S and T wave, respectively, which could be positive or negative polarity

### Evaluation and statistical analysis

Statistical analyses were performed using SPSS v26.0 (IBM, Chicago, IL, USA) and R v3.5.2 (The R Foundation for Statistical Computing). In all analyses, *p* < 0.05 was considered statistically significant. Categorical and consecutive data are presented as number (%) and mean ± standard deviation.

#### Parameter selection considering collinearity

First, all ECG parameters were translated into standardized values. We then selected from the 438 ECG parameters using two steps, considering the correlation with CA and the collinearity between the ECG parameters. For step 1, the coefficients of correlation between CA and the 438 ECG parameters were evaluated. The ECG parameters with a correlation coefficient ≥ 0.2 were selected. For step 2, from the parameters selected in step 1, the coefficient of correlation for any pairs of the parameter combinations (if the number was X then X × [X – 1] combinations) were evaluated, excluding pairs of each parameter with itself. The parameter pairs with a correlation coefficient ≥ 0.9 (defined as a ‘strong correlation’) were determined and the parameters that demonstrated the highest coefficient of correlation for CA in step 1 compared with any counterparts were selected for further analysis. Furthermore, the parameters not included in any pairs with a ‘strong correlation’ were selected for further analysis.

#### Modeling of biological age using ECG parameters

##### PCA

Biological age by PCA (BA) was modeled using ECG parameters by two steps, as previously reported [13].

1 $$ \mathrm{pre}-\mathrm{BA}=\left({\sum}_{i=1}^m{\sum}_{j=1}^n{\beta}_{ij}\left(\frac{x_j-{\overline{x}}_j}{sd\left({x}_j\right)}\right){p}_i\right), $$

where m indicates the number of principal components, i indicates their individual orders, n indicates the number of ECG parameters, j indicates their individual orders, β indicates the coefficient in the PCA, *x* indicates each ECG parameter, and ${\overline{x}}_j$ and *sd(x)* indicate the average value and the standard deviation of each ECG parameter, respectively. The *p*_*i*_ was calculated using the following formula:

2 $$ {p}_i=\left({\mathrm{R}}^2\ \mathrm{in}\ \mathrm{a}\ \mathrm{univariate}\ \mathrm{linear}\ \mathrm{regression}\ \mathrm{model}\ \mathrm{with}\ \mathrm{each}\ \mathrm{principal}\ \mathrm{component}\ \mathrm{for}\ \mathrm{CA}\right)/\left(\mathrm{sum}\ \mathrm{of}\ {\mathrm{R}}^2\ \mathrm{in}\ \mathrm{the}\ \mathrm{univariate}\ \mathrm{linear}\ \mathrm{regression}\ \mathrm{model}\mathrm{s}\ \mathrm{with}\ \mathrm{each}\ \mathrm{principal}\ \mathrm{component}\ \mathrm{for}\ \mathrm{CA}\mathrm{r}\right). $$

For step 2, BA was calculated using the following formula:

3 $$ \mathrm{BA}=\mathrm{pre}-\mathrm{BA}\times sd\left(\mathrm{CA}\right)+\overline{CA}+\left(\mathrm{CA}-\overline{CA}\right)\times \left(1-B\right), $$

where *sd* (CA) and $\overline{CA}$ indicate the standard deviation and the average value of CA, respectively, and *B* indicates the standardized coefficient in the univariate linear regression analysis in which pre-BA and CA are the dependent and independent variables, respectively.

##### KDM

Biological age by KDM algorithm (BA_E_ and BA_EC_) was modeled using ECG parameters with the following equations [14].

4 $$ {\mathrm{BA}}_E=\frac{\sum_{j=1}^m\left({x}_j-{q}_j\right)\left(\frac{k_j}{s_j^2}\right)}{\sum_{j=1}^m{\left(\frac{k_j}{s_j}\right)}^2}, $$

5 $$ {\mathrm{BA}}_{\mathrm{EC}}=\frac{\sum_{j=1}^m\left({x}_j-{q}_j\right)\frac{k_j}{s_j^2}+\frac{CA}{s_{BA}^2}}{\sum_{j=1}^m{\left(\frac{k_j}{s_j}\right)}^2+\frac{1}{s_{BA}^2}}, $$

6 $$ {r}_{char}=\frac{\sum_{j=1}^m\frac{r_j^2}{\sqrt{1-{r}_j^2}}}{\sum_{j=1}^m\frac{r_j}{\sqrt{1-{r}_j^2}}}, $$

7 $$ {S}_{BA}^2=\left(\frac{\sum_{j=1}^n\left(\right[{BA}_{Ei-{CA}_i\left]-{\sum}_{i=1}^n\left[{BA}_{Ei}-{CA}_i\right]/n\right){}^2}}{n}\right)-\left(\frac{1-{r}_{char}^2}{r_{char}^2}\right)\times \left(\frac{\Big[{CA}_{max}-{Ca}_{\mathit{\min}\Big]{}^2}}{12m}\right), $$

where *k*_*j*_ indicates the beta of an ECG parameter regressed on BA_E_, *q*_*j*_ indicates the beta of a constant regressed on BA_E_, and *s*_*j*_ indicates the root mean squared error of an ECG parameter regressed on BA_E_. However, given that BA_E_ was not measurable, the root mean squared errors from the regressions between each ECG parameter and CA (rather than BA_E_) were used [16]. The value ${r}_j^2$ indicates the variance explained by the regression of CA on *m* parameters.

#### Evaluation of the predictability of biological age for mortality

The predictive capabilities of CA, BA (by PCA), and BA_E_ and BA_EC_ (by KDM) for all-cause death and cardiovascular death were evaluated by the area under the curve (AUC) with the receiver operating curve. Patients were also divided into four CA categories of 20–39 years old, 40–59 years old, 60–74 years old, and ≥ 75 years old, and a similar evaluation was performed for each separate CA category. The comparison among CA and the biological age algorithms (BA, BA_E_, and BA_EC_) or the age categories were tested by the paired or the unpaired Delong’s test for two ROC curves [17], respectively.

## Results

### Patient characteristics

The study patients included 6897 men (53.7%) and the mean age was 55.5 ± 15.0 years. In men, the mean ages of alive and decreased patients were 54.1 ± 14.4 years and 70.9 ± 12.1 years, respectively. In women, the mean ages of alive and deceased patients were 56.9 ± 15.6 years and 70.1 ± 14.1 years, respectively. The patients’ characteristics are shown in Table S1 (see Additional file 1).

During the mean follow-up period of 320.4 days, all-cause death occurred in 55 patients (0.5 per 100 patient-years). Among the 55 all-cause death patients, 23 and 32 were cardiovascular deaths and non-cardiovascular deaths, respectively. The distributions of the deceased and alive patients are shown in Table 2.

**Table 2:** The number of deceased subjects with respect to individual CA

	Alive	All-cause death	cardiovascular death	Non-cardiovascular death
Total	12,782	55	23	32
CA				
≤ 39 years	2138	–	–	–
40–59 years	5223	11	7	4
60–74 years	4097	19	7	12
≥ 75 years	1379	25	9	16

Abbreviation: *CA* chronological age

### Parameter selection

For step 1, among the 438 ECG parameters, the correlation coefficient with CA was ≥0.2 for 71 parameters in men and for 99 parameters in women; these parameters were selected for the next step. For step 2, the coefficients of correlation were evaluated for all pairs of the parameters selected from step 1 (men: 71 × 70 = 4970 combinations; women: 99 × 98 = 9702 combinations). For both men and women, all of the parameters selected from step 1 (i.e., 71 for men and 99 for women) had combinations with a correlation coefficient ≥ 0.9. From these ECG parameters, we selected 61 parameters for men and 80 parameters for women that had a higher coefficient of correlation in step 1 compared with any counterparts. As there were no parameters not included in any pairs with a ‘strong correlation’, a total of 61 parameters for men (61 + 0) and 80 parameters for women (80 + 0) were selected for biological age modeling (Table S2; see Additional file 2).

### Construction of the biological age models

#### PCA

The PCA model was constructed using the 26 ECG parameters. The model consisted of eight unrotated principal components with corresponding eigenvalues ≥1.0. The factor loadings of the 26 ECG parameters of the PCA model are presented in Table S3 (see Additional file 3). BA by PCA ranged from − 5.31 to 132.97 (Table 3).

**Table 3:** CA and biological age estimated by three algorithms

	CA				BA				BA_E_				BA_EC_			
	Mean	SD	Min	Max	Mean	SD	Min	Max	Mean	SD	Min	Max	Mean	SD	Min	Max
Total	55.50	15.04	20.00	90.00	53.76	21.15	−5.31	132.97	44.39	26.40	−66.21	202.17	48.03	21.84	−44.94	178.58
CA of 20–39 years	32.38	5.14	20.00	39.00	24.38	9.36	−5.31	76.49	26.39	19.92	−64.43	145.85	28.24	15.75	−44.94	124.70
CA of 40–59 years	50.00	5.63	40.00	59.00	46.31	10.78	11.64	96.54	41.09	23.84	−66.21	165.13	44.08	18.40	−43.60	143.33
CA of 60–74 years	66.46	4.16	60.00	74.00	68.00	10.32	37.62	118.56	52.78	25.72	−40.22	202.09	57.23	19.34	−20.19	174.76
CA ≥75 years	79.55	3.76	75.00	90.00	85.20	11.55	47.63	132.97	59.91	27.60	−3.65	202.17	66.31	20.25	22.44	178.58

Abbreviations: *CA* chronological age, *BA* biological age by principal component analysis method, *BA*_E_ biological age by the Klemera and Doubal’s method without adjustment for chronological age, *BA*_EC_ biological age by the Klemera and Doubal’s method with adjustment for chronological age, *SD* standard deviation

#### KDM

BA_E_ and BA_EC_ were calculated using the 26 ECG parameters. The values of r_char_ and S^2^_BA_ were 8.83 and 765.39, respectively. B_AE_ ranged from − 66.21 to 202.17, whereas BA_EC_ ranged from − 44.94 to 178.58 (Table 3).

### Predictive capability of biological age for mortality

The predictive capabilities by AUC for all-cause death and cardiovascular death are shown in Table 4 and the *p*-values by the paired and unpaired Delong’s test for comparing AUCs are shown in Table S4; see Additional file 4. The AUCs for all-cause death for CA, BA, BA_E_, and BA_EC_ were 0.725, 0.731, 0.657, and 0.685 (*p*-values by the paired Delong’s test were > 0.05 for all pairs, except for 0.038 for BA vs BA_E_ and 0.002 for BA_E_ vs BA_EC_), respectively, while those for cardiovascular death were 0.674, 0.682, 0.685, and 0.692 (*p*-values by the paired Delong’s test were > 0.05 for all pairs), respectively.

**Table 4:** The predictive capability for all-cause death and cardiovascular death using CA and biological age by three algorithms

	CA			BA			BA_E_			BA_EC_		
	AUC	95% CI	*P*-value	AUC	95% CI	*P*-value	AUC	95% CI	*P*-value	AUC	95% CI	*P*-value
All-cause death												
Total	0.725	0.654–0.796	< 0.001	0.731	0.662–0.799	< 0.001	0.657	0.584–0.730	< 0.001	0.685	0.616–0.754	< 0.001
CA of 40–59 years	0.573	0.455–0.691	0.404	0.592	0.437–0.746	0.293	0.583	0.410–0.756	0.342	0.586	0.416–0.756	0.325
CA of 60–74 years	0.482	0.349–0.615	0.783	0.619	0.489–0.750	0.071	0.702	0.579–0.826	0.002	0.697	0.574–0.820	0.002
CA ≥75 years	0.680	0.563–0.797	0.001	0.575	0.477–0.673	0.197	0.534	0.414–0.655	0.556	0.539	0.421–0.658	0.498
CV death												
Total	0.674	0.556–0.791	0.003	0.682	0.556–0.807	0.002	0.685	0.568–0.802	0.002	0.692	0.573–0.811	0.001
CA of 40–59 years	0.605	0.451–0.758	0.338	0.521	0.319–0.724	0.845	0.473	0.258–0.689	0.808	0.481	0.270–0.692	0.862
CA of 60–74 years	0.549	0.282–0.817	0.653	0.673	0.452–0.894	0.113	0.811	0.704–0.918	0.004	0.803	0.686–0.921	0.005
CA ≥75 years	0.599	0.414–0.783	0.306	0.615	0.461–0.769	0.232	0.686	0.517–0.856	0.053	0.689	0.524–0.854	0.050

Abbreviations: *CA* chronological age, *BA* biological age by principal component analysis method, *BA*_E_ biological age by the Klemera and Doubal’s method without adjustment for chronological age, *BA*_EC_ biological age by the Klemera and Doubal’s method with adjustment for chronological age, *AUC* area under the curve, *CI* confidence interval

The AUCs for all-cause death and cardiovascular death according to CA categories are also shown in Table 4. Because there were no deaths in patients with a CA of 20–39 years, receiver operating curve analysis was not performed in this CA category. In patients with a CA of 40–59 years, the AUCs for all-cause death for CA, BA, BA_E_, and BA_EC_ were 0.573, 0.592, 0.583, and 0.586 (*p*-values by the paired Delong’s test were > 0.05 for all pairs), respectively, while those for cardiovascular death were 0.605, 0.521, 0.473, and 0.481, respectively (*p*-values by the paired Delong’s test were > 0.05 for all pairs). In patients with a CA of 60–74 years, the AUCs for all-cause death for CA, BA, BA_E_, and BA_EC_ were 0.482, 0.619, 0.702, and 0.697 (*p*-values by the paired Delong’s test were > 0.05 for all pairs, except for 0.006 for CA vs BA_E_ and 0.005 for CA vs BA_EC_), respectively, while those for cardiovascular death were 0.549, 0.673, 0.811, and 0.803 (*p*-values by the paired Delong’s test were > 0.05 for all pairs), respectively. In patients with a CA ≥75 years, the AUCs for all-cause death for CA, BA, BA_E_, and BA_EC_ were 0.680, 0.575, 0.534, and 0.539 (*p*-values by the paired Delong’s test were > 0.05 for all pairs, except for 0.010 for CA vs BA_EC_), respectively, while those for cardiovascular death were 0.599, 0.615, 0.686, and 0.689 (*p*-values by the paired Delong’s test were > 0.05 for all pairs), respectively. When the difference of the predictive capability among age categories was compared in a same biological age algorithm, the AUC for all-cause death by BA_EC_ was higher in patients with a CA of 60–74 years than in patients with a CA ≥75 years (unpaired Delong’s test; *p* = 0.007). The AUCs for cardiovascular death by BA_E_ and BA_EC_ were higher in patients with a CA of 60–74 years than in patients with a CA of 40–59 years (unpaired Delong’s test; *p* = 0.011 and 0.015, respectively).

## Discussion

A number of studies have examined the utility of medical records, vital signs, laboratory data [18], and epigenetic changes [19] for prediction of biological age. Differences between biological age and CA are thought to reflect acceleration of epigenetic age because of the associations with a higher risk of all-cause mortality [20, 21], CVD [19, 22], and cross-sectionally with obesity [23], earlier menopause [24], and frailty [25].

ECG can be performed easily and repeatedly, and can be analyzed instantly. ECG may be a candidate tool for estimating biological age because ECG parameters can be affected by age [5, 6]. As ECG reflects the cardiac condition, which is closely associated with the circulatory and respiratory systems, biological age estimated by ECG is suggested to reflect ‘heart age’ [10, 12]. Accordingly, when biological age estimated by ECG is utilized for predicting mortality, the prediction would primarily involve cardiovascular death. In the present study, while the AUCs for all-cause death and cardiovascular death with biological age by ECG were comparable to that with CA in total patients, the AUCs for all-cause death by biological age in patients with a CA of 60–74 years was partly higher than that with CA, and the AUCs for cardiovascular death by biological age in patients with a CA of 60–74 years showed the trend to be high. These findings suggest that biological age by ECG may provide, at least in part, a prediction of mortality related to non-cardiovascular causes. Of interest, several studies have reported that ECG can be affected by various extracardiac diseases. For example, ventricular repolarization was altered by hemodialysis [26], prolonged QTc was observed in end-stage liver disease [27, 28] and non-alcoholic fatty liver disease [29], ST segment and T waves can be altered in acute cholecystitis [30], ST depression, left ventricular hypertrophy, prolonged QTc, and T wave inversion were observed in patients with intracranial hemorrhage [31, 32], other ECG abnormalities were reported in patients with brain injury and stroke [31], and higher heart rate, prolonged QTc, and low voltage was observed in patients with thyroid dysfunction [33].

The predictive capability of biological age for various prognoses is generally considered to decline in older people because of the increased biological heterogeneity [34]. Nevertheless, in the present study the predictive capability of biological age for all-cause death and cardiovascular death was higher than that for CA in patients with a CA of 60–74 years, but was mostly comparable to CA in patients with a CA ≥75 years. However, the range of biological age in patients with a CA ≥75 years was narrower than that for patients with a CA of 60–74 years or 20–59 years, suggesting ‘decreased’ biological heterogeneity in older patients.

In the present study, we used three types of biological age assessment (BA by PCA, and BA_E_ and BA_EC_ by KDM). The distribution of BA (by PCA) was generally good, although the minimum value was lower than zero in patients with a CA of 20–39 years. By contrast, BA_E_ and BA_EC_ (by KDM) showed an extremely wide distribution, ranging from − 66.21 to 202.17 and − 44.94 to 178.58, respectively. Furthermore, for the distribution of BA_E_ and BA_EC_ in each CA category, only a BA_EC_ of ≥75 years showed a minimum value over zero. Of note, BA_E_ and BA_EC_ showed a high predictive capability for all-cause and cardiovascular death in patients with a CA of 60–74 years. Thus, despite their wide distribution, BA_E_ and BA_EC_ may be useful for predicting all-cause and cardiovascular death in patients with a CA 60–74 years.

The cost-effectiveness and non-invasive nature are major advantages of using ECG to assess biological age. We found that biological age by ECG is particularly useful for discriminating high or low risk for mortality in patients aged 60–74 years old and in discriminating the risk for cardiovascular death in patients aged ≥75 years old. Our data confirm that biological age by ECG provides an indicator of ‘heart age’. Furthermore, we provide new evidence that the predictive capability of biological age by ECG varies according to age categories.

### Limitations

There were several limitations of this study. First, all participants were patients who visited a cardiovascular hospital in an urban area. Although we analyzed the patients without structural heart diseases, they have some reasons to visit a cardiology specialized hospital, including, at least, mild symptoms or minor ECG abnormalities. Therefore, our data should be carefully interpreted and are not easily extrapolated to general populations. Second, we used the ECG parameters provided by a commercial ECG machine (GE Healthcare). Given that the approaches or algorithms used to measure the ECG waves may differ between machines from different manufacturers, revalidation with other ECG machines may be necessary. Finally, patients’ characteristics such as cardiac anatomical information, comorbidities, concomitant medications, and frailty were not included in our models.

## Conclusion

We developed a prediction model for biological age using 12-lead ECG parameters in patients without structural heart diseases. This model showed a similar predictive capability to CA for all-cause death and cardiovascular death among total patients, but partially showed a significant increase in the predictive capability among patients aged 60–74 years old.

## Supplementary Information

**Additional file 1: Table S1.** Patient characteristics.

**Additional file 2: Table S2.** Selected ECG parameters for biological age models.

**Additional file 3: Table S3.** Factor weights of the 26 ECG parameters by the PCA model.

**Additional file 4: Table S4.** Comparison of AUCs.

## Data Availability

The data cannot be shared publicly because there was no such approval in the study protocol or informed consent. Data are available from the Ethics Review Committee at the Cardiovascular Institute for researchers who meet the criteria for access to confidential data (contact via the corresponding author).

## Abbreviations

AUC: Area under the curve
CA: Chronological age
CVDs: Cardiovascular diseases
ECG: Electrocardiogram
eGFR: Estimated glomerular filtration rate
KDM: Klemera and Doubal’s method
PCA: Principal component analysis
