# Supplementary material for: Prediction of biological age and all-cause mortality by 12-lead electrocardiogram in patients without structural heart disease
Source: BMC Geriatr. 2021 Aug 11;21:460. doi: 10.1186/s12877-021-02391-8 (PMC8359578; doi:10.1186/s12877-021-02391-8)
Supplement: Supplementary file 1 — Additional file 1: Table S1. Patient characteristics. [file 12877_2021_2391_MOESM1_ESM.docx]

**Table S1. Patient characteristics**

|  | Total | Male |  |  | Female |  |  |
| --- | --- | --- | --- | --- | --- | --- | --- |
|  | n = 12837 | Total  n = 6897  (53.7%) | Alive  n = 6863  (53.7%) | Deceased  n = 34  (61.8%) | Total  n =5940  (46.3%) | Alive  n = 5919  (46.3%) | Deceased  n = 21  (38.2%) |
| Age | 55.5 ± 15.0 | 54.2 ± 14.4 | 54.1 ± 14.4 | 70.9 ± 12.1 | 57.0 ± 15.6 | 56.9 ± 15.6 | 70.1 ± 14.1 |
| BMI | 23.0 ± 3.7 | 24.2 ± 3.4 | 24.1 ± 3.4 | 23.4 ± 4.0 | 21.7 ± 3.6 | 21.7 ± 3.6 | 22.6 ± 3.4 |
| SBP | 125.8 ± 18.5 | 127.5 ± 16.7 | 127.5 ± 16.7 | 125.0 ± 16.9 | 124.0 ± 20.2 | 124.0 ± 20.2 | 131.3 ± 22.0 |
| DBP | 75.3 ± 13.8 | 77.1 ± 11.5 | 77.1 ± 11.5 | 71.0 ± 14.0 | 73.2 ± 15.8 | 73.2 ± 15.8 | 74.3 ± 10.3 |
| Heart rate | 71.1 ± 12.9 | 71.2 ± 13.5 | 71.2 ± 13.4 | 73.8 ± 17.8 | 71.0 ± 12.3 | 71.0 ± 12.3 | 74.3 ± 17.0 |
| eGFR | 74.9 ± 17.7 | 74.3 ± 17.0 | 74.5 ± 16.9 | 57.6 ± 23.0 | 75.5 ± 18.5 | 75.6 ± 18.4 | 62.7 ± 29.2 |
| LVEF | 67.8 ± 6.8 | 66.3 ± 6.6 | 66.3 ± 6.6 | 62.8 ± 13.6 | 69.5 ± 6.5 | 69.5 ± 6.5 | 66.1 ± 8.4 |
| Hypertension | 4484 (34.9) | 2628 (20.5) | 2607 (20.3) | 21 (0.2) | 1856 (14.5) | 1845 (14.4) | 11 (0.1) |
| Dyslipidemia | 2855 (22.2) | 1497 (11.7) | 1488 (11.6) | 9 (0.1) | 1358 (10.6) | 1353 (10.5) | 5 (0.0) |
| Diabetes | 923 (7.2) | 640 (5.0) | 631 (4.9) | 9 (0.1) | 283 (2.2) | 278 (2.2) | 5 (0.0) |
| Hyperuricemia | 1362 (10.6) | 1160 (9.0) | 1149 (9.0) | 11 (0.1) | 202 (1.6) | 199 (1.6) | 3 (0.0) |
| CKD | 1100 (8.6) | 617 (4.8) | 603 (4.7) | 14 (0.1) | 483 (3.8) | 475 (3.7) | 8 (0.1) |
| Anemia (Hb <11 g/dL) | 186 (1.4) | 55 (0.4) | 46 (0.4) | 9 (0.1) | 131 (1.0) | 127 (1.0) | 4 (0.0) |

Abbreviations: BMI, body mass index; SBP, systolic blood pressure; DBP, systolic blood pressure; LVEF, left ventricular ejection fraction; CKD, chronic kidney disease; Hb, hemoglobin.
