# Supplementary material for: Prediction of biological age and all-cause mortality by 12-lead electrocardiogram in patients without structural heart disease
Source: BMC Geriatr. 2021 Aug 11;21:460. doi: 10.1186/s12877-021-02391-8 (PMC8359578; doi:10.1186/s12877-021-02391-8)
Supplement: Supplementary file 2 — Additional file 2: Table S2. Selected ECG parameters for biological age models. [file 12877_2021_2391_MOESM2_ESM.docx]

**Table S2. Selected ECG parameters for biological age models**

1) Male (n = 61)

| Parameters | Step 1 | Step 2 | |
| --- | --- | --- | --- |
|  | r for CA | Having counterparts with strong correlation | Highest r for CA among the counterparts |
| P-R Interval | 0.249 | Yes | Yes |
| QTc Calculation (QTc Bazett) | 0.244 | Yes | Yes |
| P Peak Time in I | 0.146 | Yes | Yes |
| Q Peak Amplitude in I | 0.108 | Yes | Yes |
| Max R Amplitude in I | 0.101 | Yes | Yes |
| R Peak Time in I | 0.216 | Yes | Yes |
| P Duration in II | 0.121 | Yes | Yes |
| T Peak Time in II | 0.167 | Yes | Yes |
| T Duration in II | 0.104 | Yes | Yes |
| P' Peak Time in III | 0.105 | Yes | Yes |
| R' Duration in III | 0.132 | Yes | Yes |
| S Area in III | 0.185 | Yes | Yes |
| S Duration in III | 0.204 | Yes | Yes |
| Max S Amplitude in III | 0.131 | Yes | Yes |
| P Duration in aVR | 0.139 | Yes | Yes |
| QRS Area in aVR | 0.177 | Yes | Yes |
| Minimum ST level in aVR | 0.350 | Yes | Yes |
| Maximum ST level in aVR | 0.262 | Yes | Yes |
| T Peak Time in aVR | 0.202 | Yes | Yes |
| T Peak Amplitude in aVR | 0.324 | Yes | Yes |
| T Duration in aVR | 0.108 | Yes | Yes |
| P' Peak Time in aVL | 0.151 | Yes | Yes |
| P' Peak Amplitude in aVL | 0.156 | Yes | Yes |
| QRS Area in aVL | 0.219 | Yes | Yes |
| R Area in aVL | 0.195 | Yes | Yes |
| R Peak Time in aVL | 0.147 | Yes | Yes |
| Max R Amplitude in aVL | 0.218 | Yes | Yes |
| R Duration in aVL | 0.134 | Yes | Yes |
| T Peak Time in aVL | 0.101 | Yes | Yes |
| P' Peak Time in aVF | 0.104 | Yes | Yes |
| R' Duration in aVF | 0.137 | Yes | Yes |
| S Area in aVF | 0.128 | Yes | Yes |
| Max S Amplitude in aVF | 0.140 | Yes | Yes |
| P' Peak Time in V1 | 0.229 | Yes | Yes |
| P' Duration in V1 | 0.224 | Yes | Yes |
| QRS Area in V1 | 0.161 | Yes | Yes |
| P' Peak Time in V2 | 0.177 | Yes | Yes |
| P' Duration in V2 | 0.173 | Yes | Yes |
| QRS Area in V2 | 0.206 | Yes | Yes |
| R Peak Time in V2 | 0.113 | Yes | Yes |
| R Duration in V2 | 0.119 | Yes | Yes |
| T Peak Time in V2 | 0.167 | Yes | Yes |
| T Duration in V2 | 0.115 | Yes | Yes |
| P' Peak Time in V3 | 0.128 | Yes | Yes |
| P' Duration in V3 | 0.120 | Yes | Yes |
| T Peak Time in V3 | 0.189 | Yes | Yes |
| T Duration in V3 | 0.149 | Yes | Yes |
| S Area in V4 | 0.126 | Yes | Yes |
| S Duration in V4 | 0.216 | Yes | Yes |
| T Peak Time in V4 | 0.171 | Yes | Yes |
| T Duration in V4 | 0.138 | Yes | Yes |
| S Area in V5 | 0.171 | Yes | Yes |
| Max S Amplitude in V5 | 0.137 | Yes | Yes |
| S Duration in V5 | 0.210 | Yes | Yes |
| T Peak Time in V5 | 0.185 | Yes | Yes |
| T Duration in V5 | 0.114 | Yes | Yes |
| P Area (Full) in V6 | 0.138 | Yes | Yes |
| P Peak Time in V6 | 0.108 | Yes | Yes |
| S Area in V6 | 0.143 | Yes | Yes |
| S Duration in V6 | 0.134 | Yes | Yes |
| T Peak Time in V6 | 0.178 | Yes | Yes |

2) Female (n = 80)

| Parameters | Step 1 | Step 2 | |
| --- | --- | --- | --- |
|  | r for CA | Having counterparts with strong correlation | Highest r for CA among the counterparts |
| P-R Interval | 0.238 | Yes | Yes |
| QTc Calculation (QTc Bazett) | 0.142 | Yes | Yes |
| P Peak Time in I | 0.190 | Yes | Yes |
| P Duration in I | 0.155 | Yes | Yes |
| Q Peak Amplitude in I | 0.204 | Yes | Yes |
| Q Duration in I | 0.175 | Yes | Yes |
| R Area in I | 0.320 | Yes | Yes |
| Max R Amplitude in I | 0.388 | Yes | Yes |
| P Duration in II | 0.161 | Yes | Yes |
| R' Duration in II | 0.120 | Yes | Yes |
| P' Peak Time in III | 0.175 | Yes | Yes |
| P' Duration in III | 0.153 | Yes | Yes |
| R' Area in III | 0.135 | Yes | Yes |
| R' Duration in III | 0.193 | Yes | Yes |
| S Area in III | 0.219 | Yes | Yes |
| S Duration in III | 0.308 | Yes | Yes |
| Max S Amplitude in III | 0.126 | Yes | Yes |
| ST at J-point in III | 0.109 | Yes | Yes |
| Minimum ST level in III | 0.121 | Yes | Yes |
| Maximum ST level in III | 0.103 | Yes | Yes |
| P Duration in aVR | 0.186 | Yes | Yes |
| Minimum ST level in aVR | 0.141 | Yes | Yes |
| Maximum ST level in aVR | 0.107 | Yes | Yes |
| Q Peak Amplitude in I | 0.177 | Yes | Yes |
| P' Area in aVL | 0.224 | Yes | Yes |
| P' Duration in aVL | 0.213 | Yes | Yes |
| QRS Area in aVL | 0.336 | Yes | Yes |
| R Area in aVL | 0.344 | Yes | Yes |
| R Peak Time in aVL | 0.226 | Yes | Yes |
| Max R Amplitude in aVL | 0.411 | Yes | Yes |
| R Duration in aVL | 0.252 | Yes | Yes |
| P' Peak Time in aVF | 0.150 | Yes | Yes |
| P' Duration in aVF | 0.116 | Yes | Yes |
| R' Duration in aVF | 0.168 | Yes | Yes |
| S Area in aVF | 0.131 | Yes | Yes |
| Max S Amplitude in aVF | 0.164 | Yes | Yes |
| P' Peak Time in V1 | 0.278 | Yes | Yes |
| P Peak Amplitude in V1 | 0.128 | Yes | Yes |
| P' Duration in V1 | 0.280 | Yes | Yes |
| QRS Area in V1 | 0.152 | Yes | Yes |
| R Area in V1 | 0.113 | Yes | Yes |
| R' Area in V1 | 0.108 | Yes | Yes |
| Max R Amplitude in V1 | 0.161 | Yes | Yes |
| R' Duration in V1 | 0.103 | Yes | Yes |
| T Peak Amplitude in V1 | 0.181 | Yes | Yes |
| T Duration in V1 | 0.119 | Yes | Yes |
| P' Peak Time in V2 | 0.222 | Yes | Yes |
| P' Duration in V2 | 0.217 | Yes | Yes |
| QRS Area in V2 | 0.176 | Yes | Yes |
| R Area in V2 | 0.214 | Yes | Yes |
| R Peak Time in V2 | 0.171 | Yes | Yes |
| Max R Amplitude in V2 | 0.246 | Yes | Yes |
| R Duration in V2 | 0.169 | Yes | Yes |
| P' Peak Time in V3 | 0.121 | Yes | Yes |
| P' Duration in V3 | 0.114 | Yes | Yes |
| R Area in V3 | 0.242 | Yes | Yes |
| Max R Amplitude in V3 | 0.336 | Yes | Yes |
| S Area in V3 | 0.118 | Yes | Yes |
| Max S Amplitude in V3 | 0.190 | Yes | Yes |
| P Duration in V4 | 0.156 | Yes | Yes |
| Q Peak Amplitude in V4 | 0.118 | Yes | Yes |
| Q Duration in V4 | 0.137 | Yes | Yes |
| Max R Amplitude in V4 | 0.343 | Yes | Yes |
| S Area in V4 | 0.247 | Yes | Yes |
| Max S Amplitude in V4 | 0.262 | Yes | Yes |
| S Duration in V4 | 0.210 | Yes | Yes |
| P Area in V5 | 0.185 | Yes | Yes |
| P Peak Amplitude in V5 | 0.114 | Yes | Yes |
| Q Peak Amplitude in V5 | 0.188 | Yes | Yes |
| Q Duration in V5 | 0.119 | Yes | Yes |
| R Area in V5 | 0.235 | Yes | Yes |
| Max R Amplitude in V5 | 0.358 | Yes | Yes |
| S Area in V5 | 0.218 | Yes | Yes |
| Max S Amplitude in V5 | 0.205 | Yes | Yes |
| S Duration in V5 | 0.167 | Yes | Yes |
| P Peak Time in V6 | 0.134 | Yes | Yes |
| R Area in V6 | 0.144 | Yes | Yes |
| Max R Amplitude in V6 | 0.195 | Yes | Yes |
| S Area in V6 | 0.154 | Yes | Yes |
| Max S Amplitude in V6 | 0.163 | Yes | Yes |

Abbreviation: ECG, electrocardiogram; CA, chronological age.
