# Supplementary material for: Prediction of biological age and all-cause mortality by 12-lead electrocardiogram in patients without structural heart disease
Source: BMC Geriatr. 2021 Aug 11;21:460. doi: 10.1186/s12877-021-02391-8 (PMC8359578; doi:10.1186/s12877-021-02391-8)
Supplement: Supplementary file 3 — Additional file 3: Table S3. Factor weights of the 26 ECG parameters by the PCA model. [file 12877_2021_2391_MOESM3_ESM.docx]

**Table S3. Factor weights of the 26 ECG parameters by the PCA model**

| Parameters | PC1 | PC2 | PC3 | PC4 | PC5 | PC6 | PC7 | PC8 |
| --- | --- | --- | --- | --- | --- | --- | --- | --- |
| P-R Interval | -0.005 | -0.036 | 0.053 | 0.032 | 0.020 | 0.104 | -0.057 | -0.432 |
| QTc Calculation (QTc Bazett) | 0.057 | 0.004 | -0.019 | -0.048 | -0.011 | 0.222 | 0.303 | -0.165 |
| R axis | -0.048 | 0.064 | -0.165 | 0.072 | -0.028 | 0.043 | -0.057 | 0.044 |
| Maximum ST level in I | -0.089 | -0.073 | 0.036 | 0.064 | 0.087 | -0.031 | 0.022 | -0.020 |
| R Peak Time in II | 0.049 | 0.006 | -0.048 | -0.205 | -0.141 | 0.149 | 0.040 | -0.058 |
| T' Peak Amplitude in II | -0.038 | 0.106 | -0.045 | -0.042 | 0.207 | 0.047 | 0.115 | -0.112 |
| R Peak Time in III | -0.095 | -0.040 | -0.016 | -0.092 | -0.060 | 0.075 | 0.096 | 0.213 |
| Max S Amplitude in III | -0.040 | 0.069 | -0.117 | 0.059 | 0.053 | 0.088 | 0.040 | -0.070 |
| Minimum ST level in aVR | 0.037 | -0.046 | 0.165 | -0.054 | 0.065 | -0.005 | 0.145 | 0.020 |
| Maximum ST level in aVR | -0.023 | 0.086 | 0.010 | -0.111 | 0.124 | -0.005 | -0.140 | 0.178 |
| T Area in aVR | 0.089 | 0.067 | 0.000 | 0.044 | 0.050 | 0.003 | 0.064 | 0.400 |
| T Area (Full) in aVR | 0.098 | 0.089 | -0.018 | -0.022 | -0.006 | 0.008 | -0.034 | 0.220 |
| T Peak Amplitude in aVR | 0.092 | 0.074 | 0.000 | 0.033 | 0.056 | -0.003 | 0.051 | 0.358 |
| QRS Area in aVL | 0.095 | 0.069 | 0.004 | 0.127 | 0.046 | -0.123 | -0.106 | -0.163 |
| Max R Amplitude in aVL | 0.096 | 0.069 | 0.003 | 0.123 | 0.043 | -0.120 | -0.104 | -0.157 |
| Max S Amplitude in aVL | 0.044 | 0.011 | -0.060 | -0.197 | -0.153 | 0.165 | 0.063 | -0.035 |
| R Peak Time in aVF | 0.100 | 0.064 | 0.000 | 0.109 | 0.025 | -0.082 | -0.084 | -0.222 |
| P' Peak Time in V1 | 0.044 | -0.013 | 0.176 | -0.133 | 0.035 | -0.059 | 0.036 | -0.025 |
| P' Duration in V1 | 0.038 | -0.034 | 0.166 | -0.089 | 0.104 | 0.010 | 0.070 | -0.002 |
| S Area in V2 | -0.043 | 0.029 | -0.090 | 0.109 | 0.029 | 0.103 | 0.082 | 0.072 |
| Max S Amplitude in V2 | -0.041 | 0.090 | -0.099 | 0.011 | 0.115 | 0.082 | 0.047 | -0.085 |
| Maximum ST level in V3 | 0.017 | -0.012 | 0.064 | 0.024 | 0.127 | 0.384 | -0.279 | 0.034 |
| S Duration in V4 | 0.016 | -0.015 | 0.058 | 0.027 | 0.129 | 0.374 | -0.295 | 0.047 |
| Maximum ST level in V4 | -0.027 | 0.126 | 0.082 | -0.023 | -0.105 | 0.013 | 0.031 | -0.062 |
| R Peak Time in V5 | -0.025 | 0.138 | 0.074 | -0.052 | -0.117 | -0.001 | -0.035 | -0.011 |
| R Peak Time in V6 | 0.028 | -0.116 | -0.087 | -0.058 | 0.181 | -0.071 | -0.035 | 0.069 |

Abbreviation: ECG, electrocardiogram; PCA, principal component analysis.
