# Supplementary material for: Prediction of biological age and all-cause mortality by 12-lead electrocardiogram in patients without structural heart disease
Source: BMC Geriatr. 2021 Aug 11;21:460. doi: 10.1186/s12877-021-02391-8 (PMC8359578; doi:10.1186/s12877-021-02391-8)
Supplement: Supplementary file 4 — Additional file 4: Table S4. Comparison of AUCs. [file 12877_2021_2391_MOESM4_ESM.docx]

**Table S4. Comparison of AUCs**

|  | | AUC |  |  |  | P value |  |  |  |  |  |
| --- | --- | --- | --- | --- | --- | --- | --- | --- | --- | --- | --- |
|  |  | CA | BA | BA_E_ | BA_EC_ | CA vs BA | CA vs BA_E_ | CA vs BA_EC_ | BA vs BA_E_ | BA_E_ vs BA_EC_ | BA vs BA_EC_ |
| All-cause death | | | | | | | | | | | |
| AUC | Total | 0.725 | 0.731 | 0.657 | 0.685 | 0.760 | 0.141 | 0.308 | 0.038 | 0.002 | 0.110 |
|  | (A) CA 40–59 years | 0.573 | 0.592 | 0.583 | 0.586 | 0.824 | 0.921 | 0.895 | 0.858 | 0.545 | 0.900 |
|  | (B) CA 60–74 years | 0.482 | 0.619 | 0.702 | 0.697 | 0.064 | 0.006 | 0.005 | 0.079 | 0.247 | 0.092 |
|  | (C) CA ≥75 years | 0.680 | 0.575 | 0.534 | 0.539 | 0.070 | 0.102 | 0.010 | 0.405 | 0.544 | 0.266 |
| P value | (A) vs (B) | 0.333 | 0.794 | 0.291 | 0.319 |  |  |  |  |  |  |
|  | (B) vs (C) | 0.032 | 0.602 | 0.063 | 0.007 |  |  |  |  |  |  |
|  | (A) vs (C) | 0.219 | 0.866 | 0.665 | 0.227 |  |  |  |  |  |  |
| CV death | | | | | | | | | | | |
| AUC | Total | 0.674 | 0.682 | 0.685 | 0.692 | 0.775 | 0.839 | 0.706 | 0.936 | 0.534 | 0.759 |
|  | (A) CA 40–59 years | 0.605 | 0.521 | 0.473 | 0.481 | 0.469 | 0.326 | 0.340 | 0.418 | 0.273 | 0.452 |
|  | (B) CA 60–74 years | 0.549 | 0.673 | 0.811 | 0.803 | 0.319 | 0.060 | 0.061 | 0.107 | 0.333 | 0.111 |
|  | (C) CA ≥75 years | 0.599 | 0.615 | 0.686 | 0.689 | 0.851 | 0.495 | 0.469 | 0.342 | 0.801 | 0.303 |
| P value | (A) vs (B) | 0.744 | 0.359 | 0.011 | 0.015 |  |  |  |  |  |  |
|  | (B) vs (C) | 0.780 | 0.697 | 0.253 | 0.299 |  |  |  |  |  |  |
|  | (A) vs (C) | 0.964 | 0.499 | 0.156 | 0.156 |  |  |  |  |  |  |

P values for comparing AUCs among CA and three algorithms of biological age (BA, BA_E_, and BA_EC_) or among age categories were calculated by the paired or unpaired Delong's test, respectively.

AUC, area under the curve; CA, chronologic age; BA, biological age by principal component analysis method; BA_E_, biological age by the Klemera and Doubal's method without adjustment for chronological age; BA_EC_, biological age by the Klemera and Doubal's method with adjustment for chronological age; CV, cardiovascular.
